# Supplementary material for: MetaRibo-Seq measures translation in microbiomes
Source: Nat Commun. 2020 Jun 29;11:3268. doi: 10.1038/s41467-020-17081-z (PMC7324362; doi:10.1038/s41467-020-17081-z)
Supplement: Supplementary file 10 — Supplementary Data 7 [file 41467_2020_17081_MOESM10_ESM.zip › File2/Confidence_VeryHigh_Taxonomy/250782_out.krona.html]

Javascript must be enabled to view this page.

members
magnitude
magnitudeUnassigned
count
unassigned
taxon
rank

250782\_out

4

2
superkingdom
4

phylum
1239
4


SRS056519\_contig\_number\_18063
1
1263014
species

class
186801
3

3
order
186802

3
186803
family

2
189330
genus


SRS063518\_contig\_number\_contig-100\_2873.149256SRS064973\_contig\_number\_contig-100\_9086.9086
2293099
species
2


SRS144183\_contig\_number\_29364
1898203
species
1
